# Multiplexed Simultaneous High Sensitivity Sensors with High-Order Mode Based on the Integration of Photonic Crystal 1 × 3 Beam Splitter and Three Different Single-Slot PCNCs

**DOI:** 10.3390/s16071050

**Published:** 2016-07-07

**Authors:** Jian Zhou, Lijun Huang, Zhongyuan Fu, Fujun Sun, Huiping Tian

**Affiliations:** State Key Laboratory of Information Photonics and Optical Communications, School of Information and Communication Engineering, Beijing University of Posts and Telecommunications, Beijing 100876, China; zjian0304@bupt.edu.cn (J.Z.); hltnet@163.com (L.H.); 18630453127@163.com (Z.F.); sunny_fujun@163.com (F.S.)

**Keywords:** photonic crystal, resonators, filter, optical sensing and sensor, multiplexing

## Abstract

We simulated an efficient method for the sensor array of high-sensitivity single-slot photonic crystal nanobeam cavities (PCNCs) on a silicon platform. With the combination of a well-designed photonic crystal waveguide (PhCW) filter and an elaborate single-slot PCNC, a specific high-order resonant mode was filtered for sensing. A 1 × 3 beam splitter carefully established was implemented to split channels and integrate three sensors to realize microarrays. By applying the three-dimensional finite-difference-time-domain (3D-FDTD) method, the sensitivities calculated were *S*_1_ = 492 nm/RIU, *S*_2_ = 244 nm/RIU, and *S*_3_ = 552 nm/RIU, respectively. To the best of our knowledge, this is the first multiplexing design in which each sensor cite features such a high sensitivity simultaneously.

## 1. Introduction

Label-free optical sensors have recently garnered increasing interest for high-performance quantitative measurements without labeling heterogeneity introduced by fluorescent techniques. In particular, photonic crystal (PhC) sensors, because of the strong capability to manipulate light propagation, have been an attractive candidate for available label-free systems [1,2,3,4,5,6,7,8,9,10,11,12,13,14,15,16,17,18,19,20,21,22,23,24]. Recently, optical microcavities have been studied extensively for sensing. The resonant wavelength simply shifts as the environmental refractive index n*_env_* varies. Nanocavities in the one-dimensional (1D) PhC nanobeam slab confine light into an ultrasmall volume of the order of optical wavelength. In addition, a narrow slot introduced has increasingly improved the optical field localization contributing to the strong light–matter interaction [25,26,27]. The above-mentioned high-performance and miniaturized geometries enable multiple sensor microcavities to be integrated on a chip.

The integration of PhC sensors requires that multiple PhC microcavities must be positioned on one chip. Previously, Mandal et al. [6] demonstrated a nanoscale opto-fluidic sensors array based on five one-dimensional (1D) photonic crystal micro-cavities side-coupled to a bus waveguide. Although the authors of [6] presented a sensor array based on five one-dimensional (1D) photonic crystal nanobeam, the maximum sensitivity was only 130 nm/RIU. Yang et al. [21,22] demonstrated the nanoscale PhC sensors array on monolithic substrates based on a side-coupled resonant cavity array on silicon, and the sensitivities were approximately 160 nm/RIU. Caër et al. [27] demonstrated a high sensitivity of 235 nm/RIU based on an infiltrated high-Q slot photonic crystal cavity, but this sensor possessed a large footprint so that it was not suited to large-scale integration. Zou et al. [28] presented a dense microarray of 64 microcavity-based sensor nodes with series and parallel connected PhC microcavity sensors. Yan et al. [29] proposed the integration of a scheme to multiplex multi-resonance PhC cavity sensors with an additional PhC waveguide (PhCW) bandpass filter to realize a multiplexed sensor array. However, the above-mentioned references demonstrated low sensitivities or a low-site sensor array, and the combination thereof will be a promising candidate for future sensor applications.

In order to realize a high-sensitivity sensor array, the parallel connection of single-slot PhC nanobeam cavity (PCNC) sensors was simulated. The parallel connector was obtained by a 1 × 3 PhC beam splitter. Because the PCNC has several resonances in the transmission spectrum, which makes them difficult to multiplex, an additional PhC waveguide (PhCW) bandpass filter was integrated on each channel of the multiplexed sensor array to select a specific resonance. Through adjusting the taper region of PCNC and the lattice constant (*a*) of PhCW, three distinguished high-order resonant peaks appeared in the transmission spectrum. In addition, the calculated high sensitivities of the multiplexed sensor array were *S*_1_ = 492 nm/RIU, *S*_2_ = 244 nm/RIU, and *S*_3_ = 552 nm/RIU, respectively.

## 2. The 1 × 3 PhC Beam Splitter Design

**Figure 1:**
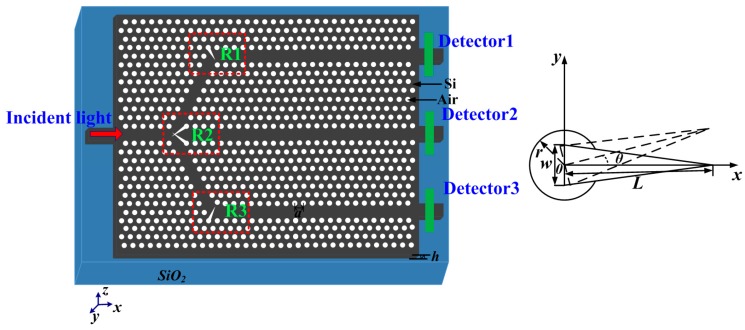
Schematic view of the 2D photonic crystal (PhC) slab 1 × 3 beam splitter lying on top of a 2-μm buried silicon oxide layer, where *a* = 460 nm, *r* = 147 nm, and *h* = 220 nm. The detailed parameters in Regions (R1, R2, and R3) are shown in the right inset, where *w* is the width of the triangle polygon, *L* is the length from the bottom to the sharp corner, and *θ* is the rotation angle versus the *x*-axis.

As a key component in photonic integrated circuits (PICs), beam splitters are indispensable. To date, a large amount of components have been designed using elementary beam splitters, including direct splitting, Y-branch [30,31], and T-branch [32,33], directional coupling splitters [34,35], 1 × 4 splitters [36], etc. In this section, we describe our design of a 1 × 3 beam splitter, and the principle was motivated by [37]. A basic PhC slab-based beam splitter was formed by a triangular lattice air cylinders etched in a 220-nm-thick silicon layer (*n_Si_* = 3.46) lying on top of a 2-μm buried silicon oxide layer (*n*_SiO2_ = 1.45), which is clearly shown in Figure 1. In this structure, the lattice constant was 460 nm, and the radius of the holes was 147 nm. The former of the beam splitter consisted of a Y branch with a 120-degree angle and a waveguide connected at the junction. The latter is composed of two 120-degree low-loss waveguide bend structures. The transmission for three branches was measured through three detectors (Dectectors 1, 2, and 3), respectively. The detailed parameters set in Regions (R1, R2, and R3) are shown in the right inset in Figure 1. The triangle polygons were introduced to reduce the reflection loss and guide the incoming wave into the branches effectively [37]. The key parameters are *w*, *L*, *θ*, and constant thickness *h* = 220 nm, where *w* is the width of the triangle polygon, *L* is the length from the bottom to the sharp corner, and *θ* is the rotation angle versus the *x*-axis.

In this work, the three-dimensional finite-difference-time-domain (3D-FDTD) method with perfectly matched layer boundary conditions was utilized for the simulations (Lumerical solutions, Inc., Vancouver, BC, Canada) [38]. Many simulations are done by modifying the parameters *L, w,* and *θ*; in this way, we obtain optimized results. The optimal parameters set were as follows: *L* = 782 nm, *w =* 220.8 nm, and *θ* = 300° (Region 1); *L* = 1150 nm, *w =* 103.04 nm, and *θ* = 210° (upper part in Region 2); *L* = 1150 nm, *w =* 103.04 nm, and *θ* = 150° (lower part in Region 2); *L* = 782 nm, *w =* 220.8 nm, and *θ* = 60° (Region 3).

**Figure 2:**
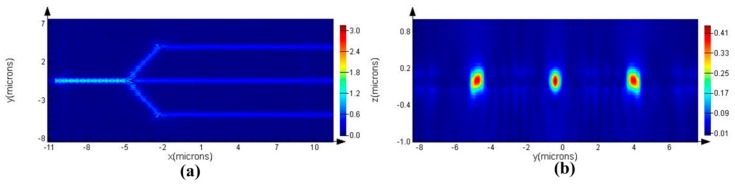
Steady-state electric field distribution for the fundamental TE-like (Transverse Electric) mode propagating through the optimized PhC slab splitter in (**a**) the *x-y* plane and (**b**) the *y-z* plane.

**Figure 3:**
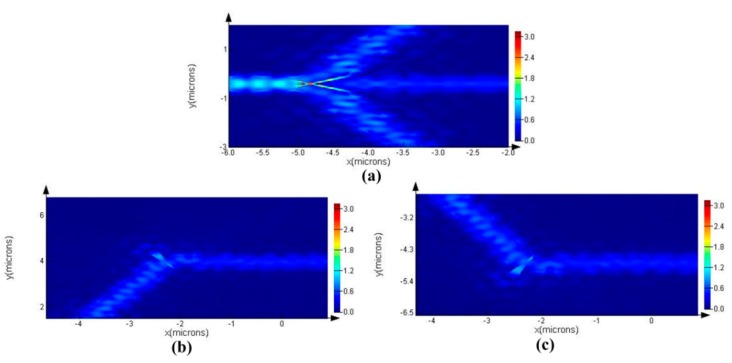
Field amplitude distributions for the optimized model corresponding to the three regions (Regions 1, 2, and 3). (**a**) Field amplitude corresponding to the Region 2; (**b**) Field amplitude corresponding to the Region 1; (**c**) Field amplitude corresponding to the Region 3.

**Figure 4:**
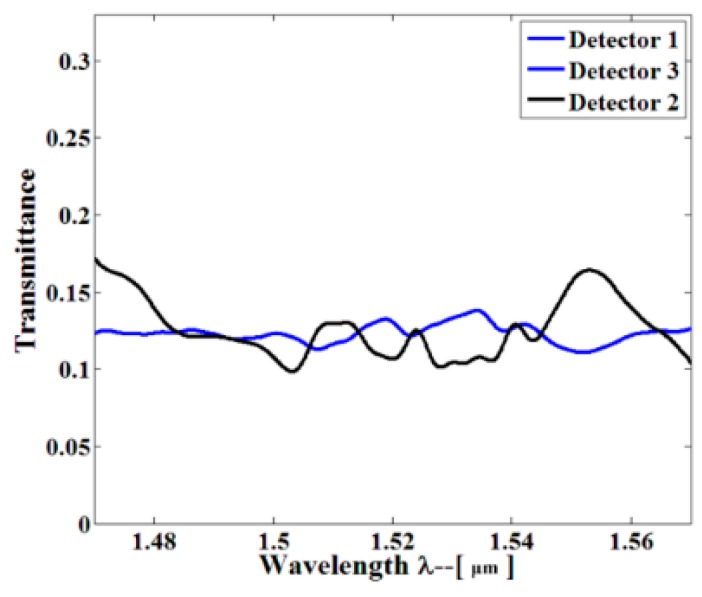
Three-dimensional finite-difference-time-domain (3D-FDTD) transmission spectra (blue and black curves) for TE-polarized light at the Detectors 1 and 3, and Detector 2 at the output of the optimum 1 × 3 beam splitter. The flat-band range is to the benefit of outstanding parallel connector.

The steady-state electric field profile is shown in Figure 2, where the excited resonant wavelength is 1491.94 nm. As clearly seen from Figure 2, the horizontal optical field was limited by a photonic band gap (PBG), and the vertical optical field was manipulated by the total internal reflection (TIR). Figure 3 shows the field amplitude of the corresponding to the red dash-line regions (Regions 1, 2, and 3) in Figure 1. As clearly shown in Figure 3, the electric field was continuous through the waveguide, while the electric field was discontinuous through the triangle polygon. This discontinuity is because of the fact that the electric displacement field is continuous across the index contrast interface. Light localization in the low index material region could be realized by the discontinuity of electric field perpendicularly to the index contrast interface. As shown in Figure 4, the flat transmission spectrum was obtained in a range from 1470 nm to 1570 nm. This flat band was able to meet the demand of parallel connectors for the sensor array.

## 3. Single-slot PCNC Design

**Figure 5:**
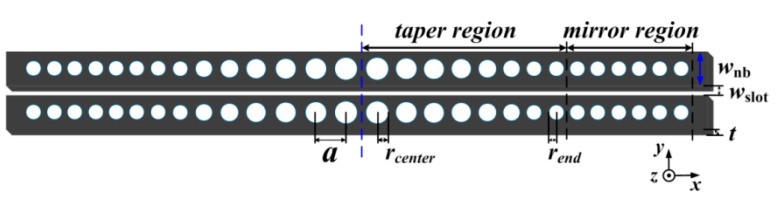
Schematics of the proposed single-slot photonic crystal nanobeam cavity (PCNC). The structure is symmetric with respect to its center (blue dashed line). The photonic mirror pitch *a* = 430 nm, the thickness *t* = 220 nm, the width *w_nb_* = 360 nm, and the slot separation *w_slot_* = 100 nm. The hole radii are parabolic tapered from *r_center_* = 120 nm in the center to *r_end_* = 93 nm on either side.

**Figure 6:**
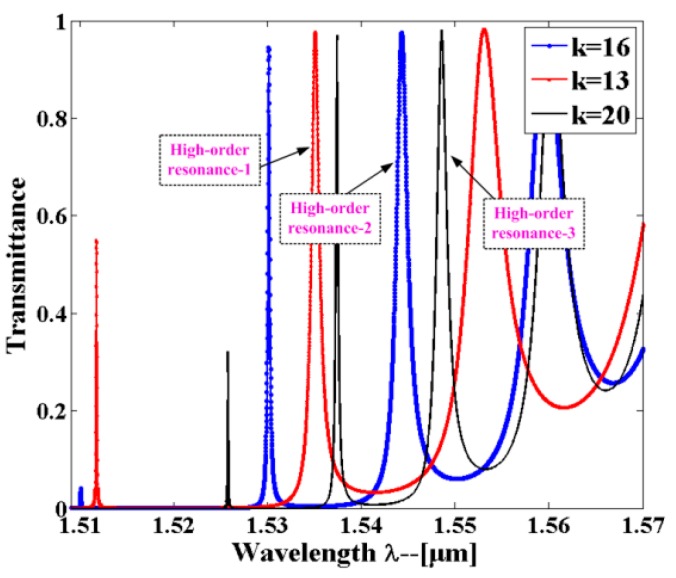
Transmission spectra of single-slot PCNC sensor from 3D-FDTD simulation. The optimized structure with *k_taper_* (13, 16, 20) mirror segments in the taper region and no additional mirrors. The background refractive index is set as RI = 1.000. The three high-order resonant peaks will be applied for parallel multiplexed sensing.

**Figure 7:**
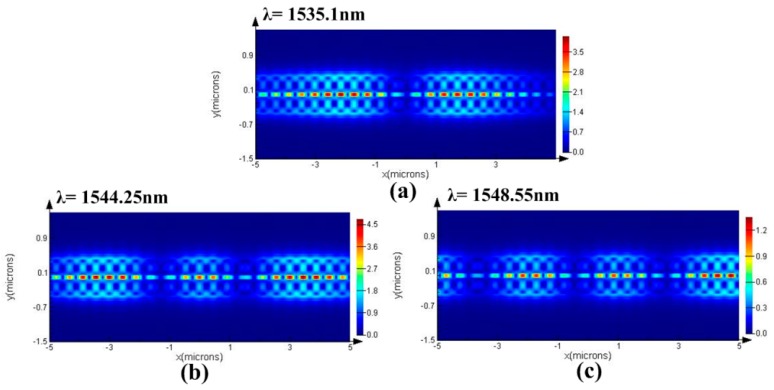
3D-FDTD simulation of the major field distribution profile in the single-slot PCNC corresponding to the three resonant modes in Figure 6. (**a**) λ = 1535.1 nm; (**b**) λ = 1544.25 nm; (**c**) λ = 1548.55 nm.

The combination of the PCNC and the air-slot waveguide results in the strong light–matter interaction [23,26], which contributes to high sensitivity. We demonstrate here that the structure consists of two parallel suspended nanobeams separated by a small air gap. The schematic of the model is shown in Figure 5. The refractive index of the silicon is *n_si_* = 3.46. The lattice constant *a* = 430 nm, width *w_nb_* = 360 nm, waveguide thickness *t* = 220 nm, and slot width *w_slot_* = 100 nm. The design principle is referred to the deterministic cavity optimized method [39]. To create the Bragg mirror, the air-holes radii are parabolic tapered from *r_center_* = 120 nm to *r_end_* = 93 nm, i.e., $r\left(n\right)=r_{center}+k^{2}\left(r_{end}-r_{center}\right)/k_{\max}^{2}$, (*k* increases from 0 to *k_max_*). Therefore, a Gaussian type confinement will be realized. In order to realize three parallel multiplexed sensing, we chose three different numbers of tapered holes but without the mirror region. To reduce the simulation time, we studied the higher order modes of a waveguide-coupled cavity possessing a moderate *Q*-factor, and the total number of *k* mirror pair segments was 13, 16, and 20, respectively. Figure 6 shows the transmission spectra obtained by exciting the input waveguide with a broadband waveguide mode source. The three separated high-order resonances will be chosen for parallel multiplexed sensing. Figure 7a–c show the major field-component distribution of the three high-order resonant modes. As expected, it can be clearly seen that the electric field profile is strongly confined within the slot of the low-index material, which contributes to the strong interaction between analytes and the cavity resonant mode.

## 4. Bandpass Filter Design

Based on the above designed sensors, in order to select three specific resonant peaks to realize a multiplexed sensor array, an additional PhCW bandpass filter was investigated. Some on-chip photonic bandpass filters, such as arrayed waveguide gratings (AWGs) and waveguide Bragg gratings (WBGs), were considered for the filters. However, WBGs are commonly long and need to operate in reflection [40]. The size of AWGs is usually too large [41] and not suitable for dense integration. In particular, the PhCW filter is a natural choice due to the sensor chip composed by PhC, leading to the easier passband alignment.

**Figure 8:**
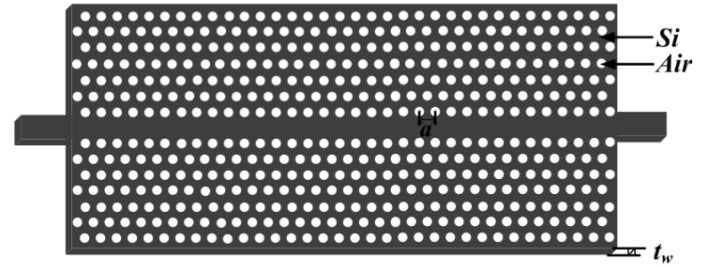
The schematic of proposed photonic crystal waveguide (PhCW) bandpss filter. The thickness is 220 nm.

**Figure 9:**
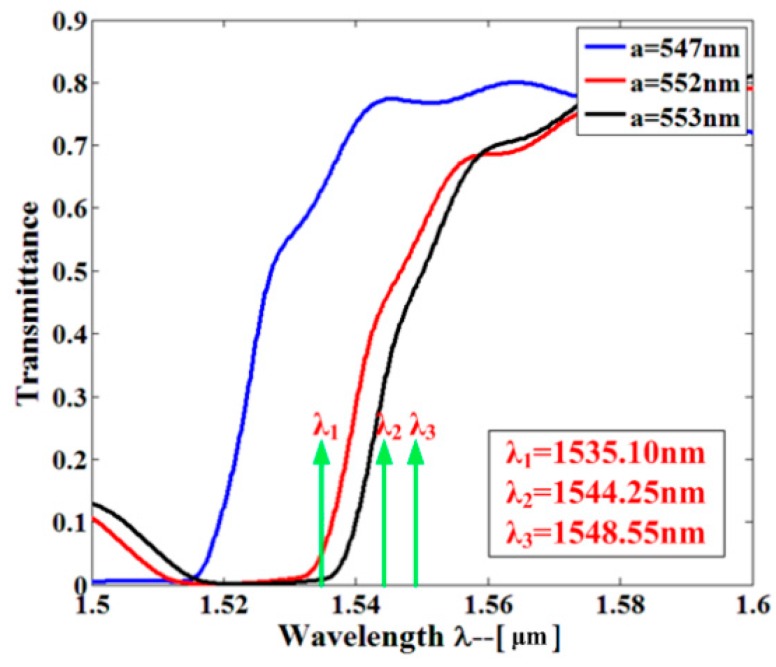
Transmission spectra of PhCW filters with different lattice constants.

A line defect in a 2D PhC lattice on a 220-nm-thick slab established a PhCW, as shown in Figure 8. In terms of the bandpass filter, light with a longer wavelength above the passband located within the bandgap of the PhC was forbidden, and shorter wavelengths outside the passband were above the light line so that the light cannot be well-confined in the waveguide. The resonant filter was created by connecting the PhCW and the sensor (Figure 5) in series. The lattice constant of the PhCW could be engineered so that a passband formed in the transmission spectrum. The PhCW filter selected the specific high-order resonance of the sensor. The filtered resonances are used for sensing when analytes are filled into the sensor. As shown in Figure 9, passbands are observed in the transmission spectra with regard to three different lattice constants (*a* = 547 nm, 552 nm, and 553 nm). The three high-order resonant modes are located within the passbands and easily filtered, as clearly observed in Figure 9.

## 5. Multiplexing Sensor Array Design

**Figure 10:**
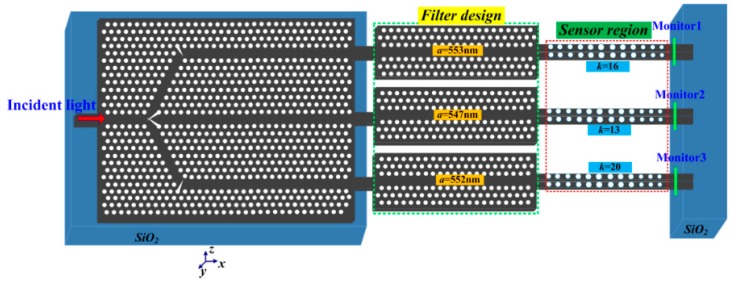
Schematic of the PhCs parallel integrated sensor array on the monolithic substrate.

**Figure 11:**
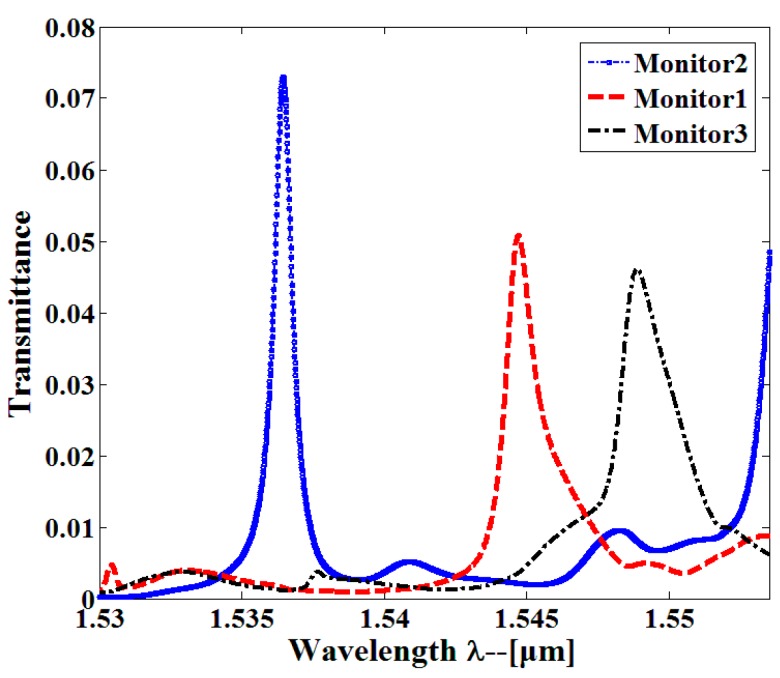
The transmission spectra of three parallel branches observed where three sensors are set in parallel connections.

Multiplexing is realized by placing arbitrary resonant cavities butt-coupled to each beam splitter branch; in this way, arbitrary resonant peaks can be obtained. The multiple resonant cavity sensors can be interrogated simultaneously on monolithic PhCs device. For proof-of-concept demonstration of the proposed PhCW filter and multiplexing, a 3-channel PhC sensor array was designed. A 1 × 3 PhC beam splitter was used to split the waveguides. Through proper engineering of the passband, three high-sensitivity multi-mode single-slot PCNC sensors could be multiplexed into a single-input and three-output system as shown in Figure 10. In each channel, the former features as a wavelength filter and the latter acts as a sensing site with a cascaded transmission band. In order to realize the sensor array, the PhCW filters were carefully employed to clearly distinguish designate high-order resonant peaks. Multiple sensors could then be interrogated simultaneously from the eventual transmission spectra. These sensors allowed different analytes to be filled into each of them. Based on the integrated structure in Figure 10, by applying the 3D-FDTD method, the output transmission spectra are calculated in Figure 11. As clearly shown in Figure 11, three resonant peaks appear in the transmission spectra.

**Figure 12:**
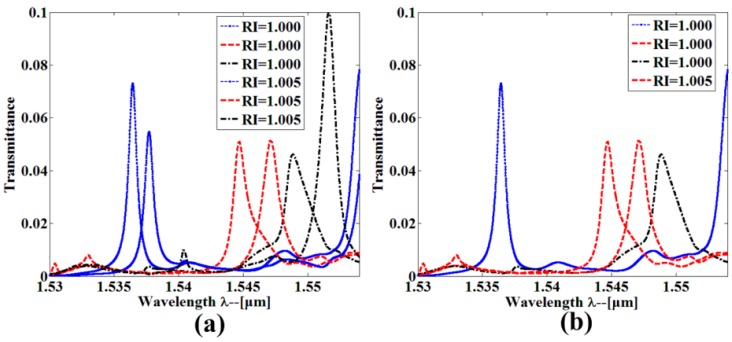
Measured transmission spectra from (**a**) three resonant wavelength shift when the refractive index of three sensors change from 1.000 to 1.005; (**b**) a resonant wavelength shift when the refractive index of one sensor change from 1.000 to 1.005, while the refractive index of the other two sensors keeps fixed.

In order to calculate sensor performance when the resonant cavities are in parallel-connections, each sensor was independently influenced by the refractive index change. Figure 12a shows the output transmission spectra of the multiplexed sensor array when three sensors are filled with refractive index RI = 1.005; three resonant peaks are obviously shifted and the resonant shift arrives at 2.46 nm, 1.22 nm, and 2.76 nm, respectively. Through the definition of the sensitivity as *S* = Δ*λ*/Δ*n*, we calculated the sensitivity of each single-slot PCNC sensor that is *S*_1_ = 492 nm/RIU, *S*_2_ = 244 nm/RIU, and *S*_3_ = 552 nm/RIU, respectively. When the refractive index of one sensor changed, the others remained fixed. As seen from Figure 12b, the resonant peak generates towards higher wavelength where the positive effect happens. This confirms that three sensors work independently. To the best of our knowledge, this is the first geometry that features such high sensitivity simultaneously, it is thus potentially an ideal platform for larger-scale high-sensitivity multiplexing refractive index-based biochemical sensing.

## 6. Conclusions

In summary, a method of a multiplexed sensor array of high-sensitivity single-slot PCNC sensor was simulated. The simultaneous measurement between a single input and three outputs was obtained by the optimized 1 × 3 beam splitter. PhCW filters were combined with single-slot PCNC sensors, contributing to the filtering of specific high-order resonant peaks to realize a large-scale sensor array. By using the 3D-FDTD simulation method, three high sensitivities were obtained. The proposed geometry is desirable for the performance of a highly parallel and label-free detection system with multiplexing capability on a monolithic substrate. Furthermore, it is more suitable for monolithic ultralow concentration detection in actual biochemical applications.
